# Adherence to the dietary approaches to stop hypertension (DASH) diet in relation to all-cause and cause-specific mortality: a systematic review and dose-response meta-analysis of prospective cohort studies

**DOI:** 10.1186/s12937-020-00554-8

**Published:** 2020-04-22

**Authors:** Sepideh Soltani, Tahereh Arablou, Ahmad Jayedi, Amin Salehi-Abargouei

**Affiliations:** 1 Department of Nutrition, School of Public Health, Iran University of Medical Sciences, Tehran, Iran; 2 Food Safety Research Center (salt), Semnan University of Medical Sciences, Semnan, Iran; 3 Department of community nutrition, School of nutritional sciences and dietetics, Tehran University of Medical Sciences, Tehran, Iran; 4 Nutrition and Food Security Research Center, Shahid Sadoughi University of Medical Sciences, Yazd, Iran; 5 Department of Nutrition, School of Public Health, Shahid Sadoughi University of Medical Sciences, PO Code, Yazd, 8915173160 Iran

**Keywords:** Dietary approaches to stop hypertension, Mortality, Cardiovascular disease, Cancer, Stroke, Dose-response analysis

## Abstract

**Background:**

Although previous investigations have proposed an association between Dietary Approaches to Stop Hypertension (DASH)-style diet and lower mortality from chronic diseases, the exposure-response relationship is not clear. The present systematic review and **meta-analysis aimed to explore the linear and non-linear dose-response** association between adherence to the DASH diet and **all-cause and cause-specific mortality**.

**Methods:**

Database search was performed in PubMed, Scopus, and EMBASE for prospective cohort studies investigating the association between adherence to the Dietary Approaches to Stop Hypertension (DASH) diet and risk of mortality. Summary hazard ratios (HRs) and 95% confidence intervals (CI) were estimated with the use of a random-effects model for the linear and nonlinear relationships. The two-stage hierarchical regression model was applied to test the potential non-linear dose-response associations.

**Results:**

The inclusion criteria were met by 17 studies (13 publications). The scores reported for adherence to the DASH diet in different studies were converted to a conventional scoring method in which the adherence score might range between 8 to 40. The linear analysis revealed that summary HRs were 0.95 (95% CI: 0.94–0.96, I^2^ = 91.6%, *n* = 14) for all-cause, 0.96 (95% CI: 0.95–0.98, I^2^ = 82.4%, *n* = 12) for CVD, 0.97 (95% CI: 0.96–0.98, I^2^ = 0.00%, *n* = 2) for stroke, and 0.97 (95% CI: 0.95–0.98, I^2^ = 63.7%, *n* = 12) for cancer mortality per each 5-point increment of adherence to the DASH diet. There was also evidence of non-linear associations between the DASH diet and all-cause and cause-specific mortality as the associations became even more evident when the adherence scores were more than 20 points (*P* < 0.005).

**Conclusion:**

Even the modest adherence to the DASH diet is associated with a lower risk of all-cause and cause-specific mortality. The higher adherence to the diet also strengthens the risk-reducing association.

**Registration:**

This review was registered in the international prospective register of systematic reviews (PROSPERO) database (registration ID: CRD42018086500).

## Introduction

Chronic diseases, including cardiovascular diseases (CVD), diabetes, cancers, and chronic respiratory diseases, are the most important causes of mortality in different communities [1]. Adherence to healthy dietary patterns is an indispensable part of clinical guidelines to prevent and control non-communicable diseases (NCDs) [2]. Dietary patterns are preferred to individual nutrients, food items, or food groups [3]; because it is proposed that dietary patterns provide a more comprehensive insight into relationships between diet and disease [4]. Furthermore, dietary patterns might be more predictive of chronic disease compared to individual foods or nutrients, because they account for possible interactions between dietary foods/nutrients in the diet [5].

In the last two decades, the Dietary Approaches to Stop Hypertension (DASH) has emerged as a healthy eating guideline [6, 7]. The diet consists of a set of recommendations including increased consumption of whole grains, fruits and vegetables, low-fat dairy products, and nuts and reduced consumption of sweets, sodium, and red and processed meats [6, 7]. The key components of the DASH diet have been related to mortality, by previous investigations; as an inverse association was represented between the intake of whole grains, fruits, vegetables, and nuts with the risk of CVD, total cancers, and mortality from all causes [8–11]; furthermore, a greater risk of all-cause and cause-specific mortality following the high consumption of red and processed meats, trans fats, simple sugars, and sodium is suggested by previous studies [12–15]. It is mentioned that the DASH diet might be a stronger predictor of disease risk compared to its components [6, 7].

The previous systematic reviews have shown that the better compliance with the DASH dietary pattern could reduce systolic and diastolic blood pressure, total cholesterol and low-density lipoprotein (LDL) [16, 17], body weight and fat [18]; and also improve glycemic control [19], and serum inflammatory markers [20]; thus, it might significantly protect against CVD, stroke, diabetes, and cancers, which are associated with a lower life expectancy rate [21]. Prospective studies on the association between the DASH diet and the mortality rate have shown some incompatible findings [22–25]. This might be because of different approaches that have been used to define the DASH diet index; as the DASH score developed by Dixon et al. [26], Günther et al. [27], and Fung et al. [28] mainly focused on dietary food groups whereas the index provided by Mellen et al. [29] was a nutrient-based dietary pattern.

Although previous meta-analyses of prospective observational studies exhibited an inverse association between adherence to the DASH diet and risk of health outcomes [30, 31], they only performed a high vs. low imitation meta-analysis, and the shape of the dose-response relationship was not determined. By determining the shape of the dose-response relationship, we will be able to show how the risk of all-cause and cause-specific mortality changes along with the greater adherence to the diet. Therefore, the present study aimed to test the linear and non-linear dose-response association between adherence to the DASH diet and the risk of CVD-, stroke-, cancer-, and all-cause mortality in the general population, by conducting a systematic review and dose-response meta-anallysis of prospective observational studies, for the first time.

## Methods

### Study protocol

We followed the Meta-Analysis of Observational Studies in Epidemiology (MOOSE) and Preferred Reporting Items for Systematic Reviews and Meta-analysis (PRISMA) guidelines for reporting the current study [32]. The review was also registered in PROSPERO (International Prospective Register of Systematic Reviews (www.crd.york.ac.uk/PROSPERO/; identifier: CRD42018086500)].

### Search strategy

Articles published up to May 3rd, 2018 in PubMed, Scopus, and Embase were searched, and two independent reviewers (SS and AJ) tried to find the relevant studies according to eligibility criteria. In case of disagreement, the principal investigator (ASA) was consulted. The following MeSH terms or text-words were used as keywords in the search strategy: (“DASH” or “dietary approaches to stop hypertension”) and (“mortality” or “fatal” or “death” or “survive” or “survival). No language or publication date restrictions were applied. Furthermore, a manual search in the reference lists of the related published papers was performed to identify other relevant references. The search was continuously updated to identify the newest relevant studies up to December 2018.

### Eligibility criteria

Studies were included in the current review if they 1) had a prospective cohort design (cohort, case-cohort, or nested case-control); 2) investigated the association between adherence to the DASH diet as an exposure and mortality from all causes, CVD, cancers, and stroke as outcome; 3) estimated the relative risk (hazard ratio, risk ratio) with the corresponding 95% confidence intervals 4) were conducted in general population who were not at a greater risk for mortality (studies who were conducted only on participants with obesity, hypertension or other conditions with greater risk for mortality were excluded). For dose-response analyses, a quantitative measure of adherence to the DASH diet for at least three quantitative categories and the total number of cases and participants/person-years or non-cases had to be available in the publication or on request from the authors. Studies that reported a linear association between the DASH diet adherence score and mortality risk were also included.

### Data extraction

Data extraction was conducted using a predefined form. The following information was extracted by two independent investigators (SS, AJ): the first author’s last name, publication year, study location, sample size, number of cases, duration of follow-up, ascertainment of outcomes, the components of the DASH diet, participants’ sex and age, and variables adjusted for in the multivariate analyses. When duplicate publications were published from the same studies, we selected the publication with the largest number of participants and complete data. The corresponding authors for studies published on Women’s Health Initiative Observational Study (WHI OS) [33] and Shanghai Men’s Health Study (SMHS) and Shanghai Women’s Health Study (SWHS) [34] were contacted to provide information concerning the ranges of the DASH diet score across categories. In one study the data only for all-cause mortality was considered in our analysis because the relevant data for cancer and CVD mortality were not provided [34].

### Assessment of the risk of bias in individual studies

The Newcastle-Ottawa scale [35] for cohort studies was used to assess the quality of included studies. Three main domains including “selection”, “comparability”, and “outcome” are considered to assess the quality in this scale. In the “selection” domain, four items are checked (representativeness of the exposed cohort, selection of the non-exposed cohort, ascertainment of exposure, and demonstration that the outcomes were not present at the start of the study). In the “comparability” domain, the control of confounders in the design or analysis of the studies is considered. The “outcome” domain, considers the outcomes ascertainment, duration of follow-up and adequacy of follow-up of cohorts. The overall quality of included studies was considered as good if they received 3–4 stars in the “selection: domain, 1-2 stars in the “comparability” domain, and 2–3 stars in the “outcome” domain. They were classified as fair if they got 2 stars in the “selection” domain and 1–2 stars in the “comparability” domain and 2–3 stars in the “outcome” domain and low if they received 0–1 star in the “selection” domain or 0 stars in the “comparability” domain or 0–1 stars in the “outcome” domain.

### Methods used to assess the adherence to the DASH diet

Four DASH diet scoring methods are used in the majority of cohort studies: 1) The traditional DASH diet scoring that was first specified by Fung et al. [28] which considers 8 components (fruits, vegetables, nuts and legumes, whole grains, low-fat dairy products, sodium, red and processed meats, and sweetened beverages). The scoring system is based on quintiles with the lowest intake receiving one point and the top quintile receiving 5 points for healthy components. The scoring for unhealthy components is reversely coded so that quintile 1 receives 5 points and quintile 5 receives one point. The overall score ranges from 8 (the lowest adherence) to 40 (the highest adherence) [28]; 2) The modified DASH score which includes 7 components: fruits; vegetables; dairy; meat, poultry, fish, and eggs; nuts, seeds, and legumes; fats and oils; and sodium, which the maximum point for each component is 10 and the possible score ranges between 0 to 70 points [34]; 3) The Mellen index, a nutrient-based index with 9 components including saturated fat, total fat, proteins, cholesterol, fiber, magnesium, calcium, potassium, sodium, that each component received 1 point; and the DASH score ranges from 0 to 9 [29]; 4) The DASH-like eating plan which includes eight dietary components: whole grains, fruits and vegetables, nuts/seeds and legumes, fish, sodium, added sugar, processed meat, and energy percentage from saturated fat. In this method the healthy intakes are assigned a score of “1,” otherwise they are scored as “0.” Points are summed across eight dietary components, leading to a total score which ranges from 0 to 8 [36]. The dietary assessment methods used by individual studies are detailed in Table 1.

**Table 1:** General study characteristics of the included studies investigating the association between adherence to DASH dirt and risk of all-cause mortality and specific mortality

Author name, publication year	Study name, country	Follow-up duration (year)	Mortality endpoints (Num)	Diet assessment method, item, assessment time	DASH dietary pattern score range/ component (score range)	Covariates
Folsom 2007 [37]	Iowa Women s Health Study, USA	16	CVD (1121)	Validated FFQ^1^, 127, baseline	11 components: Total grains, whole grains, fruits, vegetables, total dairy, total meats, nuts, seeds and dry beans, sweets, sodium, total fats, saturated fatty acids. (8–40)	Age, energy intake, education, BMI, waist/hip, smoking status and pack-years, estrogen use, alcohol intake, physical activity, and multivitamin use
			Stroke (236)			
George 2014 [22]	Women’s Health Initiative Study, USA	12.9	CVD (1483) Cancer (2384)	Validated FFQ, 122, baseline	8 components: fruit, vegetables, nuts and legumes, whole grains, low-fat dairy products, sodium, red and processed meats and sweetened beverages (8–40)	Age, race/ethnicity, educational level, marital status, smoking, physical activity, daily energy intake, postmenopausal hormone therapy, diabetes, BMI, and alcohol
Yu 2014 [34]	Shanghai Men’s Health Study, China	6.5	All-cause (4348)	Validated FFQ, 81, baseline	7 components: vegetables; fruits; dairy; meat, poultry, fish, and eggs; nuts, seeds, and legumes; fats and oils; and sodium. (0–70)	Age, Educational Attainment, Income, Cigarette smoking, Alcohol consumption, Physical activity, multivitamin supplement; BMI, Waist-to-hip ratio; History of CVD, Diabetes, or Hypertension; and Total energy intake.
			CVD (1344)			
			Cancer (1836)			
	Shanghai Women’s Health Study, China	12	All-cause (2954)	FFQ, 77, baseline		
			CVD (964)			
			Cancer (1290)			
Reedy 2014 [38]	NIH-AARP Diet and Health Study, USA	15	All-cause (54871)	Validated FFQ, 124, baseline	8 components: fruit, vegetables, nuts and legumes, whole grains, low-fat dairy products, sodium, red and processed meats, and sweetened beverages. (8–40)	Age, ethnicity, education, BMI, smoking, vigorous physical activity, energy intake, marital status, Diabetes, and alcohol
			CVD (15497)			
			Cancer (18646)			
Cuenca-García 2014 [36]	Aerobics Center Longitudinal Study, USA	11.6	All-cause (358)	3-day diet record, baseline	8 components: fruits and vegetables, fish, whole grains, nuts/seeds and legumes, sodium, added sugar, processed meat, and saturated fat. (0–8)	Age, sex, energy intake, examination year, smoking, alcohol, abnormal resting or exercise electrocardiogram, parental history of premature CVD, physical activity, and cardiorespiratory fitness
			CVD (102)			
			Cancer (134)			
Boggs 2015 [39]	Black Women s Health Study, USA	16	All-cause (1678)	Validated FFQ, 98, baseline and every 2 year	8 components: fruits (including fruit juice), vegetables, nuts and legumes, whole grains, and low-fat dairy, sodium, red and processed meats, and sugar-sweetened beverages. (8–40)	Age, total energy intake, education, marital status, vigorous exercise, television watching, smoking, BMI, and alcohol
			CVD (428)			
			Cancer (742)			
Harmon 2015 [23]	Multiethnic Cohort Study, USA	13–18	All-cause (18263)	Validated FFQ, 182, baseline	8 components: fruit, vegetables, nuts and legumes, whole grains, low-fat dairy products, sodium, red and processed meats, and sweetened beverages. (8–40)	Age, energy intake, history of diabetes, ethnicity, moderate-to-vigorous physical activity, smoking, education, marital status, hormone-replacement therapy, BMI, and alcohol
			CVD (6408)			
			Cancer (5853)			
Lassale 2016 [40]	Pan-EPIC, Europe	12.8	All-cause (15200)	Validated FFQ, baseline	8 components: fruit, vegetables, nuts and legumes, whole grains, low-fat dairy products, sodium, red and processed meats, and sweetened beverages. (8–40)	Age, sex, BMI, smoking status, physical activity level and educational level
			CVD (3761)			
			Cancer (7475)			
Park 2016 [41]	Third National Health and Nutrition Examination Survey	18.6	All-cause (296)	A single 24-h dietary recall, baseline	9 components: fruits, vegetables, nuts and legumes, low-fat dairy products, whole grains, fat (total/saturated), sodium, sweets, and red meats. (0–9)	Age, sex, race/ethnicity, educational attainment, income, smoking status, alcohol consumption, physical activity, and total energy intake
			CVD (67)			
			Cancer (79)			
Biesbroek 2017 [42]	EPIC-NL, Netherland	19.2	All-cause (3845)	Validated FFQ, 178, baseline	8 components: fruit, vegetables, nuts and legumes, whole grains, low-fat dairy products, sodium, red and processed meats, and sweetened beverages. (8–40)	Age, BMI, educational level, smoking status, total energy intake, physical activity and alcohol consumption
Zaslavsky 2017 [33]	Women s Health Initiative Study, USA	12.4	All-cause (3259)	Validate FFQ, 122, baseline and 3 year after baseline	8 components: fruit, vegetables, nuts and legumes, whole grains, low-fat dairy products, sodium, red and processed meats, and sweetened beverages. (8–40)	Age, energy and protein intake, race/ethnicity, education, income, smoking, number of frailty criteria, physical activity, and BMI
Jones 2018 [43]	EPI C-Norfo lk cohor t study, Britania	12.4	All-cause (6567)	Validated FFQ, 130, baseline	8 components: fruit, vegetables, nuts and legumes, whole grains, low-fat dairy, red and processed meats, salt, and non-milk extrinsic sugars. (8–40)	Age, sex and dietary energy, smoking status, alcohol intake, physical activity, BMI, diabetes, SES, marital status, use of antihypertensive medication, use of lipid-lowering medication and history of CVD
			CVD (1647)			
Aigner 2018 [44]	Multiethnic Cohort Study, USA	17.6	Stroke (3548)	Validated FFQ, 180, baseline	8 components: fruit, vegetables, nuts and legumes, whole grains, low-fat dairy products, sodium, red and processed meats, and sweetened beverages. (8–40)	Age, energy intake, history of diabetes, ethnicity, moderate-to-vigorous physical activity, smoking, education, marital status, hormone-replacement therapy, BMI, and alcohol

^1^Food frequency questionnaire

### Data harmonization for dose-response meta-analyses

All dietary scoring methods were converted to the conventional DASH score [28]. For studies that reported the continuous association between DASH diet score, but did not use the traditional DASH diet scale (for example, reported the result for a unit increment in the DASH diet score, ranging from 0 to 8, or 0–9, or 0–70) [34, 36, 41], we recalculated the reported risk estimates to derive hazard ratios (HRs)/ relative risks (RRs) for 5-point increment in the traditional DASH diet score (ranging from 8 to 40). For studies that did not report the continuous association and only reported the risk estimates across categories of the DASH diet score, we used the method described by Greenland and Longnecker [45, 46] to calculate study-specific HRs (linear slopes) and their corresponding 95% confidence intervals (CIs) from the natural logs of the extracted HRs (95% CIs) across adherence categories. The method needs the distribution of cases and person-years or non-cases, the median adherence score to the DASH diet, and also HRs with their 95% CIs for each DASH diet adherence categories. If the highest or lowest categories were open-ended, they were considered to have the same width as the closest category to derive the median adherence score. For studies that only reported the DASH diet score as categorical and did not use the traditional DASH diet scale (for example, used the scales of 0–8, 0–9, or 0–70) [34, 36, 41], we transformed the scores to the 32-point scale [8–40]. The study done by Aigner et al. [44], did not consider the lowest category of adherence to the DASH diet as the reference category, thus we recalculated the relative risks by using the method suggested by Hamling et al. [47]. In another study done by Jones et al. [43], the number of all-cause mortality cases for each category was not reported, and we estimated the distribution of cases across different categories according to the method developed by Aune et al. [48]. Four publications [23, 34, 38, 40], reported the results for men and women, separately; thus, their effect sizes were pooled using a random-effects model and then included in the overall meta-analysis.

### Statistical analysis

In this meta-analysis, we used HRs and 95% confidence intervals as effect size for all analyses. The reported relative risks or odds ratios in the primary studies were considered as equal as HRs. The dose-response meta-analysis consists of two parts: linear analysis and non-linear analysis. For the linear dose-response meta-analysis, assuming a linear relationship between adherence to the DASH diet and risk of all-cause and specific mortality, the HRs for each 5-point increment in adherence to the traditional DASH diet score (ranging from 8 to 40) were pooled using a random-effects model. To explore the potential non-linear relationship between adherence to the DASH diet and risk of all-cause and cause-specific mortality, the non-linear dose-response meta-analysis was performed based on the two-stage hierarchical regression model in which the difference between category-specific and reference-specific doses, expressed in quadratic terms, was calculated [49]. The spline transformation was used to estimate the within- and between-study variances [49]. All analyses were conducted using a random-effects model which takes the between-study variability into account.

The sensitivity analysis was performed to test the potential effect of each study or each dietary assessment method on pooled effect sizes. The Cochran’s Q test and I-squared (I^2^) statistic were used to evaluate the heterogeneity between studies [50]. Potential sources of heterogeneity were assessed using subgroup analyses based on gender, geographical location, length of follow-up and the number of participants. Publication bias was evaluated using Egger’s test (weighted linear regression test) and Begg’s test (rank correlation test) [51]. All analyses were conducted using Stata version 13.0. *P* < 0.05 was considered statistically significant.

### The overall quality of the meta-evidence

The quality of the present study was assessed by using the NutriGrade scoring system [52], which considers the risk of bias, study quality and limitations, heterogeneity, precision, directness, funding bias, publication bias, study design, effect size, and dose-response. The categories for the evaluation of the meta-evidence were as follows: very low (0–3.99), low (4–5.99), moderate (6–7.99), and high [4, 8, 9].

## Results

### Literature search and study characteristics

**Fig. 1:**
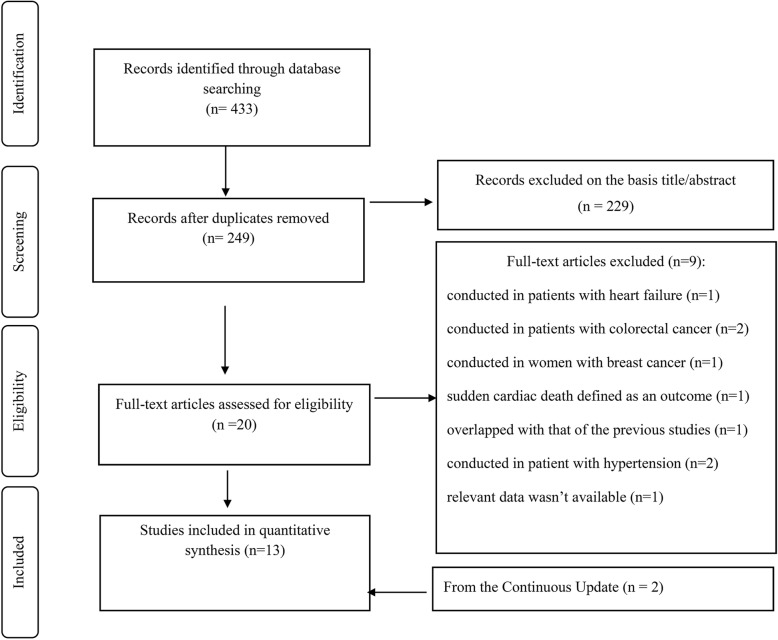
Flowchart of study selection process

In total, 433 articles were identified in our initial search; from which 184 articles were duplicates, and 229 articles did not meet the inclusion criteria after screening the titles and/or abstracts. Twenty articles were potentially relevant to be included in the systematic review. After reading their full-texts, nine articles were excluded for the following reasons: conducted on patients (*n* = 6) [patients with heart failure (*n* = 1), patients with history of colorectal (*n* = 2) and breast cancer (*n* = 1), and patients with hypertension (*n* = 2)] [25, 53–57]; sudden cardiac death was the outcome of interest (n = 1), [24], reported duplicate data [58] (*n* = 1), and not providing the sufficient data (*n* = 1) [59]. In the continuous electronic update procedure, two studies were added to eligible studies [43, 44]. The article selection procidure is provided in Fig. 1. Of the 13 related publications, one publication reported the results in two different cohort studies and therefore, was considered as two separate studies [34]. Finally, 17 prospective cohort studies (13 publications) were included in the final analyses [22, 23, 33, 34, 36–44]. Three studies (two publications) did not provide sufficient data regarding the number of deaths and risk of mortality based on each category (quartile or quintile) of the DASH diet score; thus, they were not included in the non-linear dose-response meta-analysis [34, 40].

Eleven studies were from the US [22, 23, 33, 36–39, 41, 44], four studies were from Europe [40, 42, 43], and two studies (one publication) were from Asia [34]. From 17 prospective cohort studies included in this meta-analysis: 13 studies (nine publications) reported data for all-cause mortality [23, 33, 34, 36, 38–43], 12 studies (nine publications) reported CVD mortality [22, 23, 36–41, 43], 12 studies (seven publications) reported total cancer mortality [22, 23, 36, 38–41], and two studies reported stroke mortality [37, 44]. All included studies used a validated food frequency questionnaire to assess dietary intakes except two studies that evaluated dietary intakes using 3-day dietary records [36]. All studies provided adjusted risk estimates and all of HRs were adjusted for age, smoking, energy intake and physical activity and the majority of them adjusted the associations for alcohol consumption (15 studies) [22, 23, 34, 36–39, 41–44] and BMI (16 studies) [22, 23, 33, 34, 37, 38, 40–44]. The general characteristics of the 17 eligible studies are summarized in Table 1**.**

### **Risk of bias in** individual studies

According to the Newcastle-Ottawa scale for cohort studies, all of the included studies were categorizes as good quality. All studies except two [36, 41] (which acquired 8 stars because mortality and its causes were not confirmed by a satisfactory record) received the highest possible score.

### Adherence to the DASH diet and all-cause mortality

**Fig. 2:**
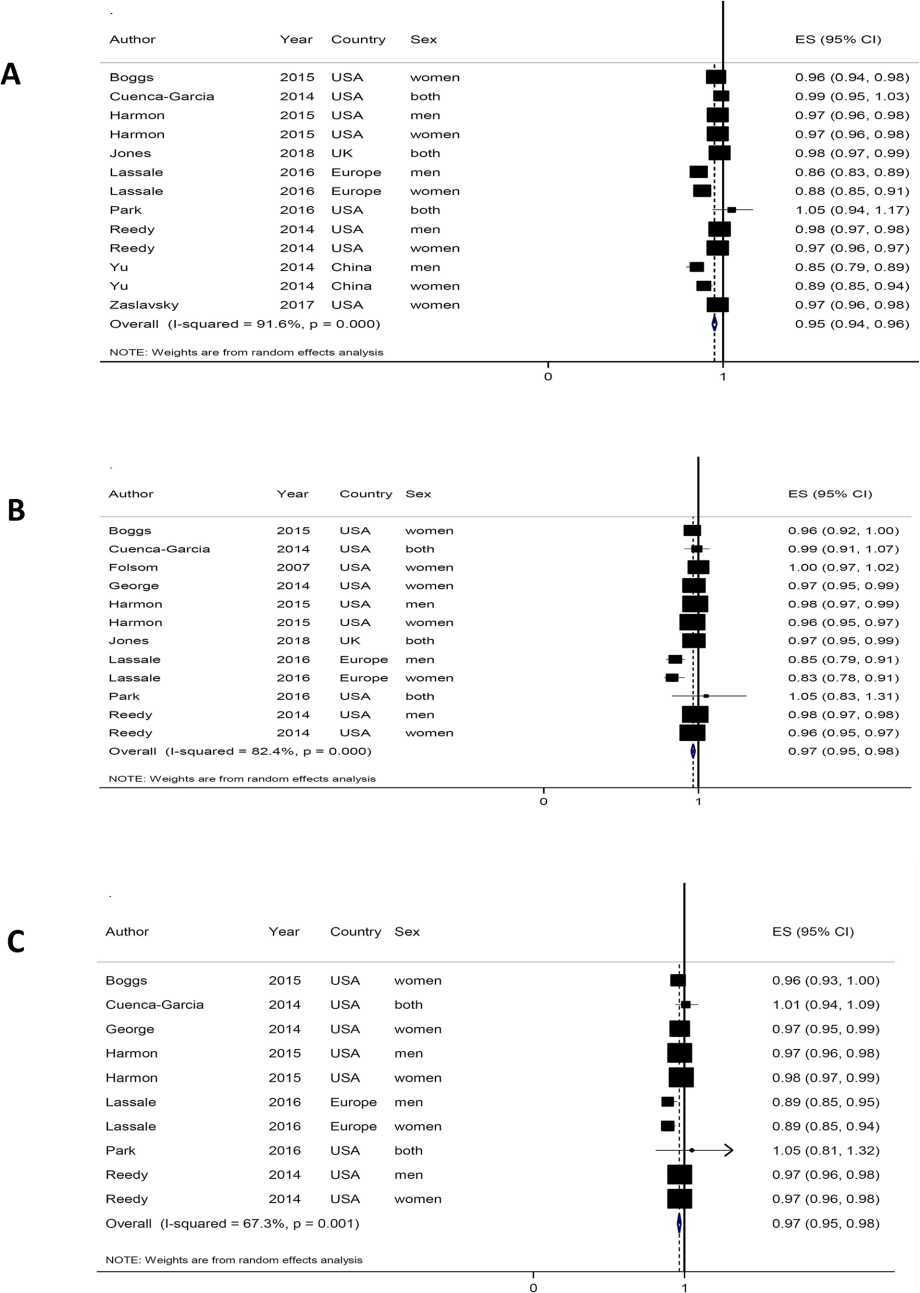
Summary of hazard ratios (HRs) of all-cause **a**, CVD **b**, and cancer **c** mortality for each 5-point greater adherence to DASH diet score; Overall estimates were calculated using random-effects model

**Fig. 3:**
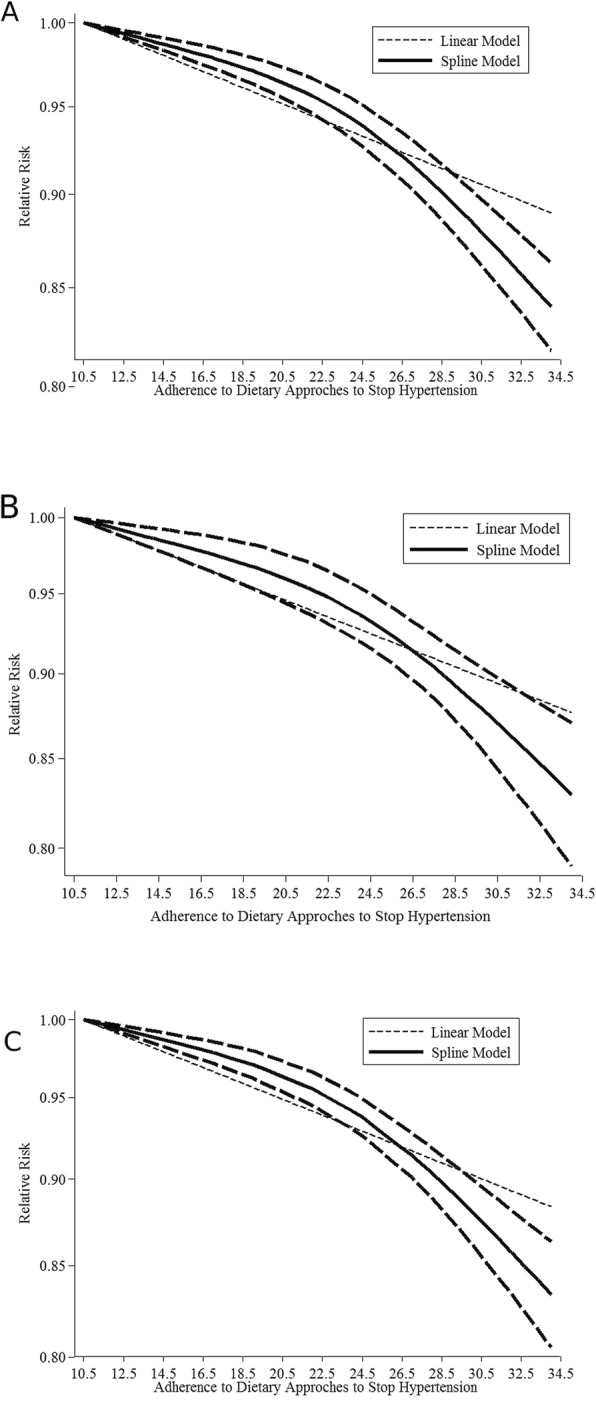
Nonlinear dose-response association between adherence to the DASH diet score and the risk of all-cause **a**, CVD **b**, and cancer **c** mortality

The relationship between adherence to the DASH diet and the risk of all-cause mortality was evaluated in thirteen cohort studies (9 Publications, comprising 1,240,308 participants) (Fig. 2a) [23, 33, 34, 36, 38–43]. The linear dose-response analysis showed that each 5-point increase in the adherence to the DASH diet is associated with 5% lower all-cause mortality (HR = 0.95, 95% CI: 0.94–0.96), and the heterogeneity was assessed to be high (I^2^ = 91.6%, *P*_heterogeneity_ < 0.001). In the sensitivity analysis, results showed that exclusion of any single comparison does not significantly alter the pooled HR (HRs ranged between 0.95 to 0.96). To further confirm the robustness of the findings, a series of sensitivity analyses were conducted by excluding the studies that used different methods to define adherence to the DASH. The similar results were obtained when we removed data addressed the Ideal Diet Index [36], Mellen index [41] and Bethesda-7 component scoring system [34]. The inverse association between adherence to the DASH diet and all-cause mortality were similar across sex, study location, study follow-up, and study participant’s characteristics. The association was more pronounced in studies with less than 13 years of follow-up and those with sample sizes greater than 100,000 participants, male population and those conducted in European countries (Supplementary Table 1). There was a slight indication of publication bias when it was checked by the use of Egger’s test, *P* = 0.02, however, it was not confirmed by Begg’s test, *P* = 0.14 (Supplementary Fig. 1A). From the eleven prospective cohort studies, ten reported the data needed for the nonlinear dose-response relationship analysis [23, 33, 36, 38, 39, 41–43]. A non-linear association was seen (P _nonlinearity_ = 0.001) with steeper inverse associations at greater adherence to the DASH diet [Fig. 3(a)].

### Adherence to the DASH diet and CVD mortality

The relationship between adherence to the DASH diet and the risk of CVD mortality was evaluated in twelve cohorts (nine publications comprising 1,314,675 participants) (Fig. 2b**)** [22, 23, 36–41, 43]. The pooled hazard risk estimation for the linear dose-response analysis was 0.97 (95% CI: 0.95–0.98) for each 5-point increment in adherence to the DASH diet, and the heterogeneity was high (I^2^ = 82.4%, P _heterogeneity_ < 0.001). Sensitivity analysis showed that the exclusion of any single study did not significantly alter the pooled HR (HRs ranged between 0.95 and 0.97). Similar results were obtained when we removed studies that used the Ideal Diet Index [36], the Mellen index [41] and the Folsom scoring system [41] to assess the adherence to the DASH diet. The significant inverse association persisted across all subgroups and the association had more strength in studies with less than 13 years of follow-up and those with sample sizes greater than 100,000 participants and conducted in European populations (Supplementary Table 2). There was no indication of publication bias using Egger’s test (*P* = 0.149) or Begg’s test (*P* = 0.451) (Supplementary Fig. 1B). Ten studies had data for the non-linear dose-response analysis [22, 23, 36–39, 41, 43]. There was evidence of non-linearity (P _nonlinearity_ = 0.006) with greater reducing associations at the highest scores of the adherence to the DASH diet (Fig. 3b).

### Adherence to the DASH diet and stroke mortality

The relationship between adherence to the DASH diet and the risk of stroke mortality was evaluated in two cohorts (193,036 participants) [37, 44]. The pooled hazard risk estimation was 0.97 (95% CI: 0.96–0.98) for each 5-point in the adherence to the DASH diet, without evidence of heterogeneity (I^2^ = 0.00%, P _heterogeneity_ = 0.563). Studies did not report the complete data for non-linear dose-response analysis.

### Adherence to the DASH diet and Cancer mortality

The relationship between adherence to the DASH diet and the risk of cancer mortality was evaluated in ten cohorts (7 Publications comprising 1,267,984 participants) (Fig. 2c) [22, 23, 36, 38–41]. The pooled hazard ratio was 0.97 (95% CI: 0.95–0.98) for each 5-point increment in the adherence to the DASH diet score, and the heterogeneity was high (I^2^ = 63.7%, P _heterogeneity_ < 0.001). The sensitivity analysis showed that further exclusion of any single study did not significantly alter the pooled HR (HRs ranged between 0.96 and 0.98). Similar results were obtained when we removed studies that used the Ideal Diet Index [36] or the Mellen index scoring system [41] from the meta-analysis. In the subgroup analysis, the study participants and the follow-up duration were found to be the possible sources of heterogeneity (Supplementary Table 3). The significant inverse association was more pronounced in studies with less than 13 years of follow-up, studies with sample sizes greater than 100,000 participants, and those conducted in the European populations (Supplementary Table 3). There was no indication of publication bias using Egger’s test (*P* = 0.207) and Begg’s test (*P* = 0.655) (Supplementary Fig. 1C). Eight prospective cohort studies provided data for the non-linear dose-response relationship analysis [22, 23, 36, 38, 39, 41]. There was evidence of nonlinearity (P _nonlinearity_ < 0.001) with steeper inverse associations at the greater adherence to the DASH diet [Fig. 3c].

### Overall quality of the meta-evidence

We rated the quality of meta-evidence for the four interested outcomes. The NutriGrade meta-evidence grading was rated as “high” for all-cause, CVD, and cancer mortality (Supplementary Table 4).

## Discussion

The present systematic review and meta-analysis showed that adherence to the DASH diet had a significant inverse dose-response association with all-cause, **CVD**, stroke, and cancer mortality. The Linear dose-response meta-analysis revealed that each 5-point increment in the adherence to the DASH diet could significantly lower the risk of all-causes, cardiovascular disease, stroke and cancer mortality by 5% (6–4%), 4% (5–2%), 3% (4–2%), and 3% (5–2%), respectively. The present results might be of clinical importance because every 5-point greater adherence to the DASH diet might prevent at least 4, 2, 2, and 2 out of every 100 all-cause, CVD-cause, and cancer-cause deaths, respectively. The non-linear dose-response analysis not only showed lower mortality rates with greater adherence to the DASH diet; but also revealed that the DASH-mortality association becomes steeper when the adherence score exceeds from 20 points (medium to high adherence).

The current study provided supportive findings that are in-line with previous meta-analyses [30, 31, 60], which found a significant inverse association between DASH diet adherence and all-cause, CVD and cancer mortality. The strengths of the current meta-analysis include the comprehensive and up to date literature searches. Also, since our analyses were performed on prospective studies, we effectively avoided recall bias and selection bias. Moreover, compared to the recent meta-analyses, the relationship between the DASH diet and all-cause, CVD, stroke, and cancer mortality were separately analyzed in a dose-response manner, for the first time.

The DASH diet is high in some of bioactive compounds (e.g., fiber, minerals, trace elements, vitamins, and phytochemicals) found in whole grains, fruits, and vegetables that have antioxidant, anti-atherogenic, anti-inflammatory, antiproliferative, and anti-tumor properties. These compounds are inversely associated with the risk of cancers, CVD, and other chronic diseases [61, 62]. On the other hand, the diet is low in harmful compounds found in processed meats, sugar-sweetened beverages, and salt, which are associated with inflammation, oxidative stress, hypertension, insulin resistance, and carcinogenesis [61]. The previous investigations have also provided evidence about the beneficial effects of the DASH diet on insulin resistance [19], hypertension [16], hyperlipidemia [53], inflammation [20], and oxidative stress [53], which are shown to be associated with CVD, stroke, and cancers.

There are a number of limitations that should be noted when interpreting our results. Although statistically significant lower mortality was observed with greater adherence to the DASH diet, a considerable heterogeneity was observed between the studies. Several subgroup analyses were performed based on gender, study duration, age, geographical location, etc.; however, none of the variables could completely explain the heterogeneity found between study results. It was shown that the effect might be different based on the geographical location of the study. The HRs were higher in the USA compared to Asia and Europe. It is proposed that the American people are different from Asian and European people with respect to lifestyle, dietary habits, and genetic background [48, 63]. The difference between DASH diet scoring methods at least in part might also explain the heterogeneity between studies. The differences in food groups considered for scoring might affect the ability of the DASH scoring methods to predict the mortality risk. For instance, only two included studies accounted for saturated fat as a component for calculating the DASH score [36, 37]; however, the other studies indirectly considered saturated fat by including components like animal foods in the scoring method. Although the studies were different in the dietary adherence assessment method, the sensitivity analysis revealed that the overall estimates did not depend on a specific method. The other source of heterogeneity between study results might be the difference in the definition of the common dietary food groups (the food groups included in different scoring methods are the same; however, the food items included to define the food groups might be different). We were not able to check this point because the majority of the included studies did not provide data about the food items included to define each food group. It should be noted that the studies included in the meta-analysis controlled the associations for the majority of potential confounders, including age, energy intake, alcohol consumption, smoking, physical activity, and body mass index; however, due to their observational nature, the results may be influenced by other uncontrolled confounding variables. Although cohort studies are less likely to be affected by recall bias, the majority of included studies used food frequency questionnaires (FFQs) to measure the participants’ dietary intakes and this tool is prone to measurement error due to misclassification. On the other hand, because the dietary assessment was performed at baseline, the dietary changes over time might also affect the observed relationship. Furthermore, only two studies in the analysis of all-cause mortality were from Asia, and all studies which provided data on CVD and cancer mortality were from western countries (mainly from the US). Therefore, more studies in other societies with different dietary habits, environmental factors, and genetic susceptibilities are needed to confirm the current results.

## Conclusion

In conclusion, the present systematic review and meta-analysis of prospective studies provided evidence regarding the dose-response association between adherence to the DASH diet and mortality from CVD, cancers, stroke, and all-causes. A greater protective association was observed among those withmedium to high adherence to the DASH diet, compared to those reporting a low adherence. Future cohort studies from different regions are recommended to replicate the current findings.

## Supplementary information

**Additional file 1: Supplementary Table 1-** Summary hazard ratio (HR) for all-cause mortality for a 5 points increment in DASH diet score. Overall estimates were calculated from random-effect models. 95% CI: 95% confidance interval; HR: hazard ratio. **Supplementary Table 2-** Summary hazard ratio (HR) for cardio vascular disease cause mortality for a 5 points increment in DASH diet score. Overall estimates were calculated from random-effect models. 95% CI: 95% confidance interval; HR: hazard ratio. **Supplementary Table 3-** Summary hazard ratio (HR) for cancer-cause mortality for a 5 points increment in DASH diet score. Overall estimates were calculated from random-effect models. 95% CI: 95% confidance interval; HR: hazard ratio. **Supplementary Table 4-** The Quality scores of a meta-analysis of adherence to DASH diet and mortality from all causes, cardiovascular disease, and cancer using NutriGrade scoring system. **Supplementary Figure 1-** Begg’s funnel plots with pseudo 95% confidence interval depicting the logarithm of hazard ratios (HRs) extracted from each study against their corresponding standard error (SE) for meta-analysis of the association between adherence to the DASH diet and all-cause (**A**), CVD-cause (**B**) and cancer-cause (**C**) mortality
