# Supplementary material for: Adherence to the dietary approaches to stop hypertension (DASH) diet in relation to all-cause and cause-specific mortality: a systematic review and dose-response meta-analysis of prospective cohort studies
Source: Nutr J. 2020 Apr 22;19:37. doi: 10.1186/s12937-020-00554-8 (PMC7178992; doi:10.1186/s12937-020-00554-8)
Supplement: Supplementary file 1 — Additional file 1: Supplementary Table 1- Summary hazard ratio (HR) for all-cause mortality for a 5 points increment in DASH diet score. Overall estimates were calculated from random-effect models. 95% CI: 95% confidance interval; HR: hazard ratio. Supplementary Table 2- Summary hazard ratio (HR) for cardio vascular disease cause mortality for a 5 points increment in DASH diet score. Overall estimates were calculated from random-effect models. 95% CI: 95% confidance interval; HR: hazard ratio. Supplementary Table 3- Summary hazard ratio (HR) for cancer-cause mortality for a 5 points increment in DASH diet score. Overall estimates were calculated from random-effect models. 95% CI: 95% confidance interval; HR: hazard ratio. Supplementary Table 4- The Quality scores of a meta-analysis of adherence to DASH diet and mortality from all causes, cardiovascular disease, and cancer using NutriGrade scoring system. Supplementary Figure 1- Begg’s funnel plots with pseudo 95% confidence interval depicting the logarithm of hazard ratios (HRs) extracted from each study against their corresponding standard error (SE) for meta-analysis of the association between adherence to the DASH diet and all-cause (A), CVD-cause (B) and cancer-cause (C) mortality [file 12937_2020_554_MOESM1_ESM.docx]

**Supplementary Table 1-** Summary hazard ratio (HR) for all-cause mortality for a 5 points increment in DASH diet score. Overall estimates were calculated from random-effect models. 95% CI: 95% conﬁdance interval; HR: hazard ratio

| **Subgroup** | | | | **Num. of**  **studies** | **Meta-analysis** | **Heterogeneity** | | | |
| --- | --- | --- | --- | --- | --- | --- | --- | --- | --- |
|  |  |  |  |  | **HR (95%CI)** | **Q statistic** | **I^2^ (%)** | ***P* _heterogeneity_** | ***P _between_*** |
| **Total** | | | | 13 | 0.95 (0.94, 0.96) | 142.25 | 91.6 | <0.001 |  |
|  | | | |  |  |  |  |  |  |
| **Sex** | **Male** | | | 4 | 0.92 (0.89, 0.96) | 85.20 | 96.5 | <0.001 | 0.008 |
| **Female** | | | | 6 | 0.95 (0.93, 0.97) | 46.05 | 89.1 | <0.001 |  |
| **Both** | | | | 7 | 0.98 (0.97, 0.99) | 1.62 | 0.00 | 0.446 |  |
|  | | | |  |  |  |  |  |  |
| **Region** | **USA** | | | 8 | 0.97 (0.96, 0.98) | 13.89 | 49.60 | 0.053 | <0.001 |
| **Europe** | | | | 3 | 0.91 (0.82, 0.99) | 85.37 | 97.7 | <0.001 |  |
| **Asia** | | | | 2 | 0.87 (0.83, 0.91) | 1.36 | 26.4 | 0.244 |  |
|  | | | |  |  |  |  |  |  |
| **Study follow-up** | | **<13 years** | | 7 | 0.92 (0.88, 0.95) | 118.99 | 95.00 | <0.001 | <0.001 |
| **>13 years** | | | | 6 | 0.97 (0.96, 0.98) | 12.75 | 60.8 | 0.026 |  |
|  | | | |  |  |  |  |  |  |
| **Study participants** | | | **<100,000** | 9 | 0.96 (0.95, 0.97) | 41.89 | 80.9 | <0.001 | 0.501 |
| **>100,000** | | | | 4 | 0.93 (0.90, 0.96) | 100.11 | 97.00 | <0.001 |  |
| ^1^ *P*-heterogeneity within subgroups with the use of a random-effects model.  ^2^ *P*-heterogeneity between subgroups with the use of a fixed-effects model. | | | | | | | | | |

**Supplementary Table 2-** Summary hazard ratio (HR) for cardio vascular disease cause mortality for a 5 points increment in DASH diet score. Overall estimates were calculated from random-effect models. 95% CI: 95% conﬁdance interval; HR: hazard ratio

| **Subgroup** | | | | **Num. of**  **studies** | **Meta-analysis** | **Heterogeneity** | | | |
| --- | --- | --- | --- | --- | --- | --- | --- | --- | --- |
|  |  |  |  |  | **HR (95%CI)** | **Q statistic** | **I^2^ (%)** | ***P* _heterogeneity_** | ***P _between_*** |
| **Total** | | | | 12 | 0.96 (0.95, 0.98) | 62.66 | 82.4 | <0.001 | -- |
| **Sex** | | | |  |  |  |  |  |  |
|  | **Male** | | | 3 | 0.97 (0.94, 0.99) | 17.93 | 88.8 | <0.001 | <0.001 |
| **Female** | | | | 6 | 0.96 (0.94, 0.98) | 25.64 | 80.5 | <0.001 |  |
| **Both** | | | | 6 | 0.97 (0.95, 0.99) | 0.64 | 0.00 | 0.727 |  |
| **Region** | | | |  |  |  |  |  |  |
|  | **USA** | | | 9 | 0.97 (0.96, 0.98) | 27.18 | 70.6 | 0.001 | 0.005 |
| **Europe** | | | | 3 | 0.89 (0.78, 0.99) | 27.56 | 92.7 | <0.001 |  |
| **Study follow-up** | | | |  |  |  |  |  |  |
|  | | **<13 years** | | 5 | 0.93 (0.88, 0.97) | 30.71 | 87.00 | <0.001 | 0.024 |
| **>13 years** | | | | 7 | 0.97 (0.96, 0.98) | 26.83 | 77.6 | <0.001 |  |
| **Study participants** | | | |  |  |  |  |  |  |
|  | | | **<100,000** | 8 | 0.97 (0.96, 0.98) | 13.82 | 49.4 | 0.054 | 0.443 |
| **>100,000** | | | | 4 | 0.93 (0.90, 0.96) | 48.25 | 93.8 | <0.001 |  |
| ^1^ *P*-heterogeneity within subgroups with the use of a random-effects model.  ^2^ *P*-heterogeneity between subgroups with the use of a fixed-effects model. | | | | | | | | | |

**Supplementary Table 3-** Summary hazard ratio (HR) for cancer-cause mortality for a 5 points increment in DASH diet score. Overall estimates were calculated from random-effect models. 95% CI: 95% conﬁdance interval; HR: hazard ratio

| **Subgroup** | | | | **Num. of**  **studies** | **Meta-analysis** | **Heterogeneity** | | | |
| --- | --- | --- | --- | --- | --- | --- | --- | --- | --- |
|  |  |  |  |  | **HR (95%CI)** | **Q statistic** | **I^2^ (%)** | ***P* _heterogeneity_** | ***P _between_*** |
| **Total** | | | | 10 | 0.97 (0.95, 0.98) | 27.53 | 67.3 | 0.001 | -- |
| **Sex** | | | |  |  |  |  |  |  |
|  | **Male** | | | 3 | 0.96 (0.95, 0.98) | 9.64 | 79.3 | 0.008 | 0.378 |
| **Female** | | | | 5 | 0.96 (0.95, 0.98) | 15.85 | 74.8 | 0.003 |  |
| **Both** | | | | 5 | 1.00 (0.94, 1.08) | 0.09 | 0.00 | 0.768 |  |
| **Region** | | | |  |  |  |  |  |  |
|  | **USA** | | | 8 | 0.97 (0.97, 0.98) | 4.74 | 0.00 | 0.692 | <0.001 |
| **Europe** | | | | 2 | 0.89 (0.86, 0.92) | 0.00 | 0.00 | 1.000 |  |
| **Study follow-up** | | | |  |  |  |  |  |  |
|  | | **<13 years** | | 4 | 0.94 (0.88, 0.99) | 18.61 | 83.9 | <0.001 | 0.023 |
| **>13 years** | | | | 6 | 0.97 (0.97, 0.98) | 3.72 | 0.00 | 0.591 |  |
| **Study participants** | | | |  |  |  |  |  |  |
|  | | | **<100,000** | 6 | 0.97 (0.97, 0.93) | 3.99 | 0.00 | 0.551 | 0.113 |
| **>100,000** | | | | 4 | 0.95 (0.92, 0.97) | 21.03 | 85.7 | <0.001 |  |
| ^1^ *P*-heterogeneity within subgroups with the use of a random-effects model.  ^2^ *P*-heterogeneity between subgroups with the use of a fixed-effects model. | | | | | | | | | |

**Supplementary Table 4-** The Quality scores of a meta-analysis of adherence to DASH diet and mortality from all causes, cardiovascular disease, and cancer using NutriGrade scoring system.

| Mortality | No of studies | RR (95%CI) | I^2^ (%) | Nutri-Grade Score |
| --- | --- | --- | --- | --- |
| All-cause mortality | 13 | 0.95 (0.94, 0.96) | 91.6 | 8 (High) |
| CVD mortality | 12 | 0.96 (0.95-0.98) | 82.4 | 8 (High) |
| Cancer mortality | 10 | 0.97 (0.95-0.98) | 63.7 | 8 (High) |
| Stroke mortality | 2 | 0.97 (0.96-0.98) | 0.00 | 4 (Low) |

**C**

**B**

**A**

**Supplementary Figure 1-** Begg’s funnel plots with pseudo 95% confidence interval depicting the logarithm of hazard ratios (HRs) extracted from each study against their corresponding standard error (SE) for meta-analysis of the association between adherence to the DASH diet and all-cause (**A**), CVD-cause (**B**) and cancer-cause (**C**) mortality
